# Current Landscape and Future Direction of PD-1/PD-L1 Checkpoint Inhibitors in Cancer Treatment

**DOI:** 10.3390/biom13081209

**Published:** 2023-08-01

**Authors:** Simona Serratì, Francesca Margheri

**Affiliations:** 1 Laboratory of Experimental Pharmacology, IRCCS Istituto Tumori Giovanni Paolo II, 70124 Bari, Italy; 2 Department of Experimental and Clinical Biomedical Sciences, University of Florence, 50134 Florence, Italy

Immune checkpoints are involved in controlling the activation or inhibition of the immune response and are associated with receptors on the immune cell surface [1]. A type of immunotherapy is represented by Immune Checkpoint Inhibitors (ICIs) which have the function of stimulating the anti-tumor immune response by blocking the cell surface receptors of T lymphocytes [2]. The blockade of PD-1/PD-L1 and CTLA-4 molecules represents two of the most promising checkpoint inhibition strategies used in recent years [3]. In particular, PD-1 plays a crucial inhibitory role in the signalling of programmed cell death in response to the T cell-mediated process [4]. It is widely expressed in different types of immune cells within the tumor microenvironment (TME). During the binding of PD-1 to its ligand PD-L1, an inhibitory signal is transmitted resulting in T-cell inhibition and, ultimately, exhaustion [5]. PD-1 acts primarily to limit T cell activity in peripheral tissues in the later stages of tumor progression, unlike CTLA-4 which instead regulates T cell activity in the early stages of tumor growth [6].

Even if ICIs are a rapidly evolving type of immunotherapy, there are many unresolved issues that may limit their use and efficacy. Examples are situations in which the tumor does not respond to the first-time administration of therapy (primary resistance), or cases in which the therapy becomes ineffective after the first stages of response (acquired resistance), as has been observed in 30% of patients with melanoma, which responds well at the start of treatment, then develops resistance acquired during the course of therapy [7].

Therefore, despite observation of long-lasting responses detected in a small portion of cancer patients, the majority of patients demonstrated non-responses or may have undergone resistance despite previous tumor remission [8,9]. Although the molecular mechanisms of ICIs resistance have not been completely elaborated, it is generally accepted that ICIs resistance is attributable to the interplay among tumor intrinsic mechanisms, tumor microenvironment (TME) and host-related factors [10]. Possible causes include low or no expression of PD-L1, tumor mutational burden, and epigenetic modifications, alterations in crucial signaling pathways, and gut bacterial species in hosts [11]. An in-depth and comprehensive exploration into the resistance mechanisms is critically important for the development of therapeutic approaches to overcame resistance and to boost ICI efficacy.

Along with the limitations of resistance, another important event requiring attention in the use of ICIs is the occurrence of immune-related adverse events (irAEs). These side effects can manifest early or late during the immunotherapy treatment and occur in different spectrums and grades [12]. The irAEs can be different depending on the type of immunotherapy, patient’s susceptibility as well as the site of the tumor. Toxicity can be systemic, dermatological, gastrointestinal and endocrine, although the skin and colon are the most frequently affected organs [13]. Recent data from a multicenter study on the spectrum and grade of irAEs demonstrated that late irAEs (i.e., after 12 months of ICI treatment) are quite common in long responders and they occur with different manifestations [12]. These results underline the importance of monitoring the evolution of toxicity over time.

Due to the complexity of resistance mechanisms and the broad spectrum of inflammatory toxicities for which there is not yet a reliable biomarker that predicts or correlates with adverse events, it becomes increasingly urgent to implement drug combination strategies to overcome ICI resistance and identify biological markers able to predict the disease progression and specific toxicities for the selection of personalized therapeutic options. In this regard, extracellular vesicles (EVs) and exosomes have emerged as role players in contribution to resistance and as carriers of drugs in cancer therapy. In particular, exosomal PD-L1 binding to T cell surface PD-1 is able to deliver inhibitory signals [14]. Evidence in tumor-bearing mice indicated that exosome elimination mitigates tumor burden and enhances the potency of anti-PD-1/PD-L1 antibodies [15]. These findings suggest that EV modulation may be a viable and necessary concomitant therapy for anti-PD-1/PD-L1 antibody potentiation. Considering EVs as nanocarriers of various bioactive molecules, the potential to cross biological barriers, achieve good biocompatibility, avoid immunogenicity, stability, and good safety profile have made them ideal candidates for targeted drug delivery in addition to biomarker and vaccine applications. Although EVs and exosomes are promising drug delivery machinery, their clinical applications are limited mainly due the lack of appropriate scalable isolation methods in addition to requirement for efficient drug loading technologies [16]. Future efforts should be undertaken to translate these issues into clinical application, thus rendering the notion of “from bench to bedside” possible.

## References

[1] Esfahani K., Roudaia L., Buhlaiga N., Del Rincon S.V., Papneja N., Miller W.H. (2020). A Review of Cancer Immunotherapy: From the Past, to the Present, to the Future. Curr. Oncol..

[2] Shiravand Y., Khodadadi F., Kashani S.M.A., Hosseini-Fard S.R., Hosseini S., Sadeghirad H., Ladwa R., O’byrne K., Kulasinghe A. (2022). Immune Checkpoint Inhibitors in Cancer Therapy. Curr. Oncol..

[3] Seidel J., Otsuka A., Kabashima K. (2018). Anti-PD-1 and Anti-CTLA-4 Therapies in Cancer: Mechanisms of Action, Efficacy, and Limitations. Front. Oncol..

[4] Riella L.V., Paterson A.M., Sharpe A.H., Chandraker A. (2012). Role of the PD-1 Pathway in the Immune Response. Am. J. Transplant..

[5] Amarnath S., Mangus C.W., Wang J.C.M., Wei F., He A., Kapoor V., Foley J.E., Massey P.R., Felizardo T.C., Riley J.L. (2011). The PDL1-PD1 Axis Converts Human T_H_1 Cells into Regulatory T Cells. Sci. Transl. Med..

[6] Dermani F.K., Samadi P., Rahmani G., Kohlan A.K., Najafi R. (2019). PD-1/PD-L1 immune checkpoint: Potential target for cancer therapy. J. Cell. Physiol..

[7] Zaretsky J.M., Garcia-Diaz A., Shin D.S., Escuin-Ordinas H., Hugo W., Hu-Lieskovan S., Torrejon D.Y., Abril-Rodriguez G., Sandoval S., Barthly L. (2016). Mutations Associated with Acquired Resistance to PD-1 Blockade in Melanoma. N. Engl. J. Med..

[8] Chen P.-L., Roh W., Reuben A., Cooper Z.A., Spencer C.N., Prieto P.A., Miller J.P., Bassett R.L., Gopalakrishnan V., Wani K. (2016). Analysis of Immune Signatures in Longitudinal Tumor Samples Yields Insight into Biomarkers of Response and Mechanisms of Resistance to Immune Checkpoint Blockade. Cancer Discov..

[9] Ribas A., Hodi F.S., Kefford R., Hamid O., Daud A., Wolchok J.D., Hwu W.-J., Gangadhar T.C., Patnaik A., Joshua A.M. (2014). Efficacy and safety of the anti-PD-1 monoclonal antibody MK-3475 in 411 patients (pts) with melanoma (MEL). J. Clin. Oncol..

[10] Zhang C., Zhang C., Wang H. (2023). Immune-checkpoint inhibitor resistance in cancer treatment: Current progress and future directions. Cancer Lett..

[11] Bagchi S., Yuan R., Engleman E.G. (2021). Immune Checkpoint Inhibitors for the Treatment of Cancer: Clinical Impact and Mechanisms of Response and Resistance. Annu. Rev. Pathol. Mech. Dis..

[12] Nigro O., Pinotti G., De Galitiis F., Di Pietro F.R., Giusti R., Filetti M., Bersanelli M., Lazzarin A., Bordi P., Catino A. (2020). Late immune-related adverse events in long-term responders to PD-1/PD-L1 checkpoint inhibitors: A multicentre study. Eur. J. Cancer.

[13] Marin-Acevedo J.A., Chirila R.M., Dronca R.S. (2019). Immune Checkpoint Inhibitor Toxicities. Mayo Clin. Proc..

[14] Chen G., Huang A.C., Zhang W., Zhang G., Wu M., Xu W., Yu Z., Yang J., Wang B., Sun H. (2018). Exosomal PD-L1 contributes to immunosuppression and is associated with anti-PD-1 response. Nature.

[15] Xie F., Xu M., Lu J., Mao L., Wang S. (2019). The role of exosomal PD-L1 in tumor progression and immunotherapy. Mol. Cancer.

[16] Vader P., Mol E.A., Pasterkamp G., Schiffelers R.M. (2016). Extracellular vesicles for drug delivery. Adv. Drug Deliv. Rev..

